# Trapdoor spiders of the genus *Cyclocosmia* Ausserer, 1871 from China and Vietnam (Araneae, Ctenizidae)

**DOI:** 10.3897/zookeys.643.10797

**Published:** 2017-01-06

**Authors:** Xin Xu, Chen Xu, Fan Li, Dinh Sac Pham, Daiqin Li

**Affiliations:** 1 College of Life Sciences, Hunan Normal University, Changsha, Hunan, China; 2 Centre for Behavioural Ecology and Evolution (CBEE), College of Life Sciences, Hubei University, Wuhan, Hubei, China; 3 Department of Biological Sciences, National University of Singapore, 14 Science Drive 4, Singapore 117543; 4 Graduate University of Science and Technology, Vietnam Academy of Science and Technology, 18 Hoang Quoc Viet, Cau Giay, Hanoi, Vietnam

**Keywords:** China, Cyclocosmia, taxonomy, trapdoor spider, Vietnam

## Abstract

A species of the genus *Cyclocosmia* Ausserer, 1871 collected from Guizhou Province, China is diagnosed and described as new to science: *Cyclocosmia liui* Xu, Xu & Li, **sp. n.** (♀). New records of *Cyclocosmia latusicosta* Zhu, Zhang & Zhang, 2006 (♀) from China (Yunnan Province) and Vietnam (Vinh Phuc Province, Ninh Binh Province), and *Cyclocosmia ricketti* (Pocock, 1901) collected from Jiangxi Province, China are also reported in this study.

## Introduction

The mygalomorph family Ctenizidae is ancient, long-lived, regionally endemic and dispersal-limited, and thus is of long-standing and persistent conservation significance in many regions of the world (Zhu et al. 2006; Opatova et al. 2013, 2016). Ctenizids are widely distributed in east and southeast Asia, north and south America, the Mediterranean region, southern Africa and Australia (World Spider Catalog 2016). These medium-sized, ground-dwelling spiders usually construct silk-lined burrows underground, which open to the surface with a trapdoor. Trapdoors covered with a layer of leaf litter and/or a sheet of moss match the background well, thus making them very difficult to spot in the field (Gertsch and Wallace 1936; Gertsch and Platnick 1975; Hunt 1976; Bond and Coyle 1995).

Despite being present across much of the world, Ctenizidae is represented by only approximately 130 extant species-level taxa (World Spider Catalog 2016). These taxa are conventionally divided into nine genera and two subfamilies, Ctenizinae and Ummidiinae (Ortiz 2007). Ummidiinae includes three genera, *Conothele* Thorell, 1878, *Hebestatis* Simon, 1903 and *Ummidia* Thorell, 1875. *Hebestatis* was recently removed from this subfamily since it posseses lateral sternal sigilla and a less pronounced and glabrous dorsal saddle on the tibia III, but lacks curvy spines, tarsal clavate trichobothria and centrally sclerotized spermathecae (Decae 2010). The taxonomic position of *Hebestatis* therefore remains unclear (but see Garrison et al. 2016). The subfamily Ctenizinae contains six genera, *Bothriocyrtum* Simon, 1891, *Cteniza* Latreille, 1829, *Cyrtocarenum* Ausserer, 1871, *Cyclocosmia* Ausserer, 1871, *Latouchia* Pocock, 1901, and *Stasimopus* Simon, 1892. However, this grouping currently lacks the support of any identified synpomorphies (Raven 1985).

**Figure 1.**
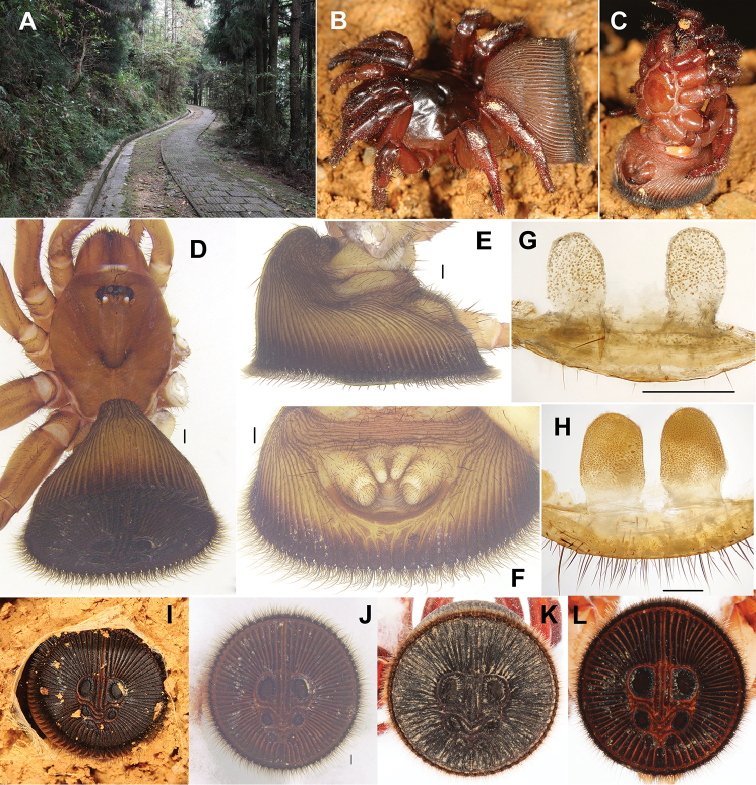
*Cyclocosmia ricketti* (Pocock, 1901) **A** habitat **B–D** habitus of female (XUC-2013-013) **E** abdomen, lateral view **F** posterior portion of opisthosoma, ventral view, showing spinnerets **G** female genitalia (XUC-2013-013), dorsal view **H** female genitalia (tianzishan-2016), dorsal view **I** showing opisthosomal disc in plugging position (XUC-2013-013) **J–L** abdomen, caudal view (**J** XUC-2013-013 **K** Tianzishan-2016 **L** C-2016-001). Scale bars 0.5 mm.

Despite being a small genus, *Cyclocosmia* contains some of the most fascinating spiders in the world (Buchli 1969; Gertsch and Platnick 1975). Their abdomens are abruptly truncated and finish in a hard, heavily sclerotized disc that is enhanced by a series of raised ribs separated by narrow grooves (Gertsch and Platnick 1975). This acts as a distinctive morphological defence to cope with intruders, such as predators, into the borrow. When the spider retreats head-first into its burrow, the abdominal disc fits tightly against the round walls of the burrow and forms an impenetrable false trapdoor (Gertsch and Wallance 1936; Gertsch and Platnick 1975). *Cyclocosmia* spiders usually build their burrows in steeply sloping banks of sandy clay (Fig. 1A). The trapdoor is usually made of silk mixed with soil and covered with a layer of leaf litter and/or moss. Like many other ctenizids, *Cyclocosmia* spiders are very difficult to find in the field because the remarkably effective camouflage of their trapdoors. Therefore, they are often regarded as one of the rarest spiders (Gertsch and Wallance 1936; Gertsch and Platnick 1975; Zhu et al. 2006).

*Cyclocosmia* is currently represented by seven nominal species: two in the USA (*Cyclocosmia torreya* Gertsch & Platnick, 1975 and *Cyclocosmia truncata* (Hertz, 1841)), one in Mexico and Guatemala (*Cyclocosmia loricata* (C. K. Koch, 1842)) and four in East and Southeast Asia (*Cyclocosmia latusicosta* Zhu, Zhang & Zhang, 2006 and *Cyclocosmia ricketti* (Pocock, 1901) in China, *Cyclocosmia lannaensis* Schwendinger, 2005 in China and Thailand, *Cyclocosmia siamensis* Schwendinger, 2005 in Thailand and Laos) (World Spider Catalog 2016). In this study, three *Cyclocosmia* species collected from China and Vietnam are diagnosed and described, one of which is believed to be new to science. One of the species is a new record from China (Yunnan) and Vietnam, based on the morphology of female specimens. Ideally, both male and female specimens would be included in the description of new species; however, it is extremely difficult to obtain adult males of trapdoor spiders. Indeed, we were unable to obtain male *Cyclocosmia* specimens in this study. By searching and digging burrows, we obtained a few immature and/or adult female specimens. Males are short-lived and leave the burrow immediately after they reach maturity to search for females (Haupt and Shimojana 2001; Haupt 2003). Therefore, collecting males is only possible at certain times of the year, and therefore is not feasible during each field trip.

## Materials and methods

Specimens were studied using an Olympus SZX16 stereomicroscope. Anatomical details were examined and photographed with on Olympus BX51 compound microscope and a Canon 7D camera. Genitalia were cleared in boiling KOH for a few minutes to dissolve soft tissues. All the specimens were deposited at the Centre for Behavioural Ecology and Evolution (CBEE), College of Life Sciences, Hubei University, Wuhan, China. All lengths are given in millimetres. Leg and palp measurements are given in the following order: total length (femur + patella + tibia + metatarsus + tarsus).

Abbreviations used:

ALE
 anterior lateral eye 


AME
 anterior median eye 


PLE
 posterior lateral eye 


PME
 posterior median eye 


MOA
 median ocular area 


PMS
 posterior median spinneret 


PLS
 posterior lateral spinneret 


## Taxonomy

### Genus Cyclocosmia

Taxon classificationAnimaliaAraneaeCtenizidae

Ausserer, 1871

Cyclocosmia Ausserer, 1871, type species Cyclocosmia truncata (Hentz, 1841): 144.
Chorizops Ausserer, 1871, type species by original designation Actinopus loricatus C. L. Koch, 1842, synonymised by Gertsch & Platnick, 1975: 15.

#### Diagnosis.

The genus *Cyclocosmia* differs from all the other genera of Ctenizidae by the abruptly truncated abdomen forming a heavily sclerotized disc that is enhanced by a series of raised ribs and grooves (Gertsch and Platnick 1975) (Fig. 1B-D, I-L). Genera *Galeosoma* and *Idiosoma* of the family Idiopidae have the similar abdominal form as *Cyclocosmia*, but the genus *Galeosoma* can be distinguished from *Cyclocosmia* by the distinctly truncated abdomen without ribs or grooves, and the genus *Idiosoma* can be distinguished from *Cyclocosmia* by the moderately truncated abdomen, even though with ribs or grooves. Moreover, the arrangement of eyes is also different, *Cyclocosmia* with two eye rows, yet the two genera of Idiopidae with three eye rows (Gertsch and Platnick 1975; Zhu et al. 2006).

### Cyclocosmia ricketti

Taxon classificationAnimaliaAraneaeCtenizidae

(Pocock, 1901)

Fig. 1

Halonoproctus ricketti Pocock, 1901: 209, pl. 21, f. 1 (described female of Pocock, 1901 was not examined).
Cyclocosmia ricketti Simon, 1903: 887, f. 1044–1047; Gertsch & Platnick, 1975: 18, f. 28–29, 32, 36; Song, Zhu & Chen, 1999: 36, f. 16H, K–L; Schwendinger, 2005: 227, f. 2–8, pl. 1D; Zhu, Zhang & Zhang, 2006: 120, f. 1, 2A–E; Zhang, Gao & Li, 2007: 385, f. 101; Yin et al., 2012: 134, f. 13a–e.

#### Material examined.

Female (XUC-2013-013), Mt. Nan, Ciping Town, Jinggangshan City, Jiangxi Province, China, 26.56892°N, 114.16350°E, 22 October 2013, collected by F.X. Liu, X. Xu and C. Xu; 1 Juvenile (C-2016-001), Cemetery of Jinggangshan Revolutionary Martyrs, Ciping Town, Jinggangshan City, Jiangxi Province, 26.57873°N, 114.15960°E, 31 August 2016, collected by X. Xu; 1 female (Tianzishan-2016), Mt. Tianzi, Zhangjiajie, Hunan Province, China, 29.40°N, 110.44°E, 10 March 2016, collected by S.F. Peng.

#### Diagnosis.

*Cyclocosmia ricketti* differs from other species of *Cyclocosmia* by the character of 23-33 radiating ribs on each side of abdominal disc (Fig. 1I-L), and parallel-sided spermathecae (Fig. 1G, H). It can be distinguished from *Cyclocosmia latusicosta* by the lack of the elevated central zone inside the upper pair of muscle impressions (Fig. 1I-L). More details see Zhu et al. 2006.

#### Description.

Female (XUC-2013-013). Total length, including chelicerae, 14.00; carapace 6.50 long, 5.40 wide; abdomen 6.50 long, 9.30 wide. Carapace red-brown and smooth, with a few marginal hairs in the front of ocular area, four long bent bristles in longitudinal row running through ocular area, the posterior two bristles have been damaged (Fig. 1D). Ocular area with a black ring around each eye of the anterior eye row and a black band in front of fovea. Cervical groove and radial furrows distinct. Fovea deep and procurved, U-shaped, its greatest width occupying one fourth of carapace width at that point. Eyes set on low tubercle, ocular 0.70 long, 1.70 wide anteriorly, 1.70 wide posteriorly. Clypeus height 0.60. Anterior eye row straight and posterior eye row recurved, both rows almost equal in length. Ratio of eyes, ALE: AME: PLE: PME (0.40: 0.25: 0.30: 0.20). ALE-AME 0.25, AME-AME 0.20, PLE-PME 0.10, PME-PME 0.70. MOA 0.70 long, 0.70 wide in front, 1.10 wide at back. Chelicerae red-brown, inner margin with eight teeth and six denticles, outer margin with seven teeth and four denticles. Rastellum raised on prominent angled projection and consisting of many short black teeth. Labium yellow-brown, 1.10 long, 1.20 wide, with three black cuspules anteriorly. Maxilla yellow-brown, 2.30 long, 1.50 wide, with a few black cuspules at base.

Legs yellow-brown. Tibiae and tarsi of pedipalps, and distal three segments of legs I and II with numerous horn-like spines, metatarsus III with a few short dorsal and two ventrally spines, tarsus III with a few dark spines prolaterally and ventrally. Legs each with three tarsal claws, paired claws with a single large tooth, unpaired claw lacking tooth. Palp with a single claw bearing one tooth. Measurements: palp 10.06 (4.00 + 2.10 + 2.30 + 2.20), leg I 11.00 (4.20 + 1.60 + 2.50 + 1.50 + 1.20), leg II 8.60 (3.20 + 1.40 + 1.50 + 1.40 + 1.10), leg III 8.50 (3.50 + 1.30 + 1.20 + 1.00 + 1.50), leg IV 12.20 (4.00 + 2.60 + 2.10 + 2.00 + 1.50). Formula: 4123.

Abdomen funnel-shaped and dark yellow-brown (Fig. 1D). Caudal disc slightly concave, 8.80 in transversal diameter and 8.50 in longitudinal diameter, with two rids running dorso-ventrally and 32/33 (XUC-2013-013), 29/28 (Tianzishan-2016), 33/33 (C-2016-001) radiating ribs on each side. Abdominal disc with six well-marked muscle impressions (Fig. 1I-L). Four spinnerets, with inner pair small and one-segmented, and outer pair slightly longer and three-segmented (Fig. 1F). Genitalia with paired spermathecae (Fig. 1G, H), sack-like, parallel-sided, the length of each one is more or less one and a half times its width: length = 0.55 mm, width = 0.33 mm (XUC-2013-013); length = 1.17 mm, width = 0.78 mm (Tianzishan-2016).

#### Distribution.

China (Fujian, Hunan, Jiangxi, Zhejiang, Sichuan).

#### Remarks.

*Cyclocosmia ricketti* was diagnosed and described based on the holotype female collected from Fujian, and since then, according to the character of the abdominal disc with 23-33 ribs on each side, researchers have identified specimens collected from Hunan, Zhejiang, Sichuan as *Cyclocosmia ricketti*. Here, a specimen collected from Jiangxi is also identified as *Cyclocosmia ricketti* on the basis of this character; *Cyclocosmia ricketti* was not recorded in Jiangxi before. Males remain unknown.

### Cyclocosmia latusicosta

Taxon classificationAnimaliaAraneaeCtenizidae

Zhu, Zhang & Zhang, 2006

Fig. 2

Cyclocosmia latusicosta Zhu, Zhang & Zhang, 2006: 121, f. 4, 5A–D, 6A–J (described female of Zhu, Zhang & Zhang, 2006 was not examined); Zhang, Gao & Li, 2007: 385, f. 1–100.

#### Material examined.

Female (17-1-2013), Tam Dao Town, Tam Dao National Park, Vinh Phuc Province, Vietnam, 21.45847°N, 105.64834°E, 17 January 2013, collected by D. Li, F.X. Liu and X. Xu; Female (XUC-2016-017), Cuc Phuong National Park, Nho Quan, Ninh Binh Province, Vietnam, 20.34915°N, 105.59927°E, 31 May 2016, D. Li, F. Li and F.X. Liu; 3 females and 1 juveniles (LH-2016-(002-005)), rubber plantation, Hekou City, Yunnan, 22.537°N, 103.942°E, 15 September 2016, F. Li, F.X. Liu and L. Yu.

#### Diagnosis.

Females of *Cyclocosmia latusicosta* are different from other *Cyclocosmia* species by their abdominal disc with 22–27 wide ribs on each side, each upper muscle impression with an elevated zone connected to the outer rim of each upper muscle impression, and the length of spermathecae being more or less 1.7–2.0 times longer than width.

#### Description.

**Figure 2.**
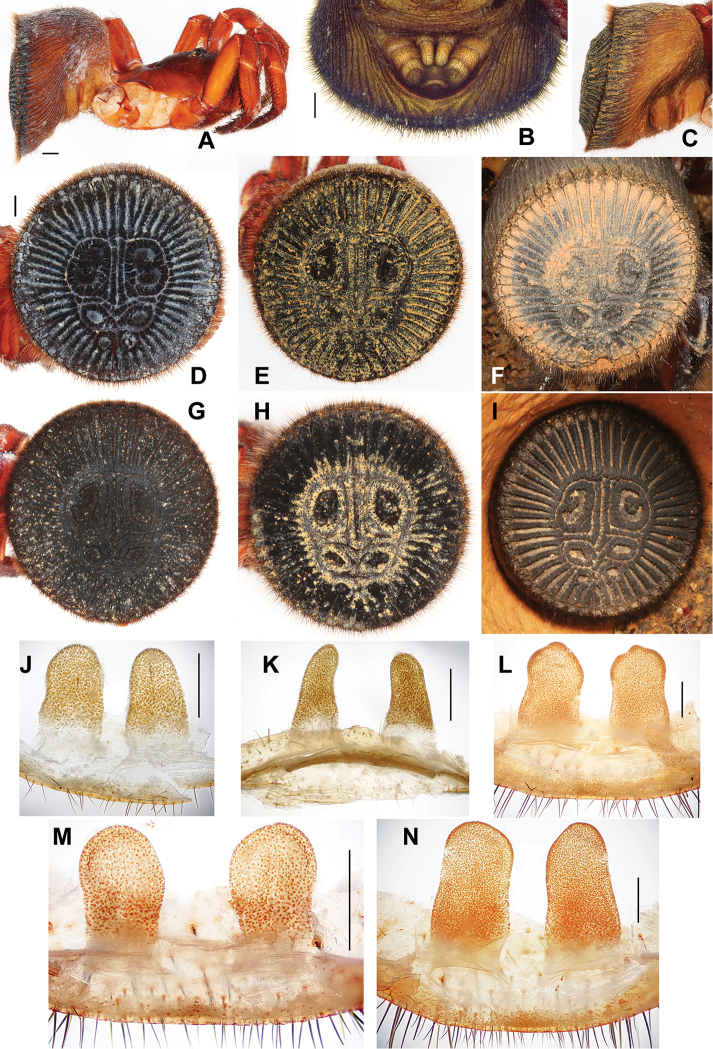
*Cyclocosmia latusicosta* Zhu, Zhang & Zhang, 2006 **A** habitus of female, lateral view (17-I-2013) **B** posterior portion of opisthosoma, ventral view, showing spinnerets **C** abdomen, lateral view (XUC-2016-017) **D–I** abdomen, caudal view (**D** 17-I-2013 **E** XUC-2016-017 **F** LH-2016-002 **G** LH-2016-003 **H** LH-2016-004 **I** LH-2016-005) **J–N** female genitalia (**J** 17-I-2013 **K** XUC-2016-017 **L** LH-2016-003 **M** LH-2016-004 **N** LH-2016-005), dorsal view. Scale bars **A, B, D** 1mm, **J–N** 0.5 mm.

Female. Total length, including chelicerae, 17.10–30.20; chelicerae 2.60–4.90 long; carapace 7.50–14.50 long, 6.71–12.50 wide; abdomen 9.30–11.60 long, 12.20–18.50 wide. Carapace red-brown and smooth, with a few marginal hairs and a long bristle in front of ocular area, six long bent bristles in longitudinal row and two bristles in latitudinal running through ocular area (Fig. 2A). Ocular area black, with a black band in front of fovea and beside ocular area respectively. Cervical groove and radial furrows distinct. Fovea deep and procurved, U-shaped, its greatest width occupying one fifth of carapace width at that point. Eyes set on low tubercle, ocular 0.90 long, 2.00 wide anteriorly, 2.00 wide posteriorly, occupying one fourth of carapace width at that point. Clypeus height 2.40. Both anterior and posterior eye rows straight and almost equal in length. Ratio of eyes, ALE: AME: PLE: PME (0.38: 0.25: 0.20: 0.20). ALE-AME 0.28, AME-AME 0.28, PLE-PME 0.02, PME-PME 0.80. MOA 0.80 long, 0.78 wide in front, 1.20 wide at back. Chelicerae red-brown, inner margin with six teeth and one denticle, outer margin with seven teeth and four denticles. Rastellum raised on prominent angled projection and consisting of many short black teeth. Labium yellow-brown, 1.40 long, 1.40 wide, with three black cuspules anteriorly. Maxilla yellow-brown, 15.90 long, 1.20 wide, with a few black cuspules at base.

Legs yellow-brown. Tibiae and tarsi of pedipalps, and distal three segments of legs I and II with numerous horn-like spines, metatarsus III with a few short dorsal and ventrally spines, tarsus III with a few dark spines prolaterally and ventrally. Legs each with 3 tarsal claws, paired claws with a single large tooth, unpaired claw lacking tooth. Palp with a single claw bearing one tooth. Measurements: palp 12.10 (4.20 + 1.80 + 3.10 + 3.00), leg I 14.00 (5.00 + 2.00 + 3.00 + 2.00 + 2.00), leg II 11.20 (4.10 + 2.00 + 2.00 + 1.50 + 1.60), leg III 11.00 (4.10 + 1.00 + 2.20 + 1.70 + 2.00), leg IV 15.20 (5.00 + 3.00 + 2.50 + 2.50 + 2.20). Formula: 4123.

Abdomen funnel-shaped and dark yellow-brown (Fig. 2A). Caudal disc slightly convex, 11.00 in transversal diameter and 10.20 in longitudinal diameter, with two rids running dorso-ventrally (with small interrupt at the groove outer the upper pair muscle impressions) and 24/25 (17-1-2013), 23/25 (XUC-2016-017), 24/23 (LH-2016-002), 28/26 (LH-2016-003), 23/24 (LH-2016-004), 22/23 (LH-2016-005) radiating ribs on each side (Fig. 2D-I). Abdominal disc with six well-marked muscle impressions. All rims within the muscle impression zone with distinct granular structures in different sizes (Fig. 2D-I). Four spinnerets, with inner pair small and one-segmented, and outer pair longer and three-segmented. Paired spermathecae sack-like, parallel-sided, each one with a length 1.7-2.0 times its width (Fig. 2J-N), length = 0.86 mm, width = 0.43 mm (17-1-2013), length = 0.94 mm, width = 0.39 mm (XUC-2016-017), length = 1.42 mm, width = 0.83 mm (LH-2016-003), length = 0.73 mm, width = 0.40 mm (LH-2016-004), length = 1.67 mm, width = 0.83 mm (LH-2016-005).

#### Distribution.

China (Guangxi, Yunnan), Vietnam (Vinh Phuc, Ninh Binh).

#### Remark.

*Cyclocosmia latusicosta* was diagnosed based on the holotype female collected from Guangxi Province, China, near the border to Vietnam. This study provides a new record from China (Yunnan) and Vietnam. We preliminarily treated the differences in the spermathecae among five specimens (17-1-2013, XUC-2016-017, LH-2016-003/004/005) as the intraspecific variation according to the work of Zhang et al. (2007). Males remain unknown.

### Cyclocosmia liui

Taxon classificationAnimaliaAraneaeCtenizidae

Xu, Xu & Li sp. n.

http://zoobank.org/BAD72BC4-CC48-44DF-BA1A-138BAAB915B5

Fig. 3

#### Holotype.

Female (C-XUX-2015), Mt. Fanjing, Taiping Town, Jiangkou County, Tongren City, Guizhou Province, China, 27.8513°N, 108.7779°E, 25 May 2015, collected by Z.Q. Li, F.X. Liu and M. Yan.

#### Etymology.

The specific name is taken from the family name of the collector Fengxiang Liu, who joined all collecting trips and has worked on spiders for a few decades.

#### Diagnosis.

**Figure 3.**
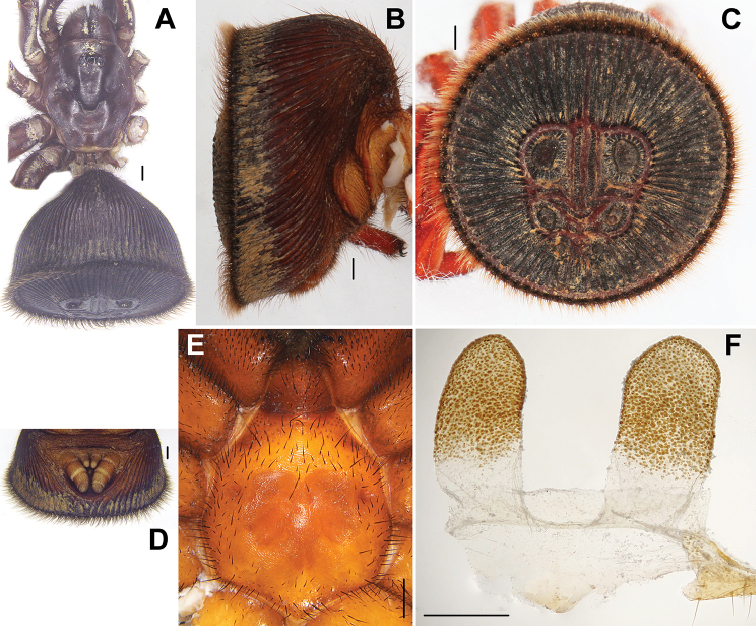
*Cyclocosmia liui* Xu, Xu & Li sp. n. **A** habitus of female (C-XUC-2015) **B** abdomen, lateral view **C** abdomen, caudal view **D** posterior portion of opisthosoma, ventral view, showing spinnerets **E** sternum, ventral view **F** female genitalia, ventral view. Scale bars **A–E** 1 mm, **F** 0.5 mm.

Female of *Cyclocosmia liui* sp. n. can be distinguished from *Cyclocosmia ricketti* by abdominal disc with 33/34 ribs on each side, the rims of the upper pair muscle impressions with distinct granular structures in almost same size, the groove around the six well-marked muscle impressions dark red colour, and the middle pair muscle impressions with an elevated central zone connected to the inner rim of muscle impression (Fig. 3C). It is similar to *Cyclocosmia latusicosta* in the shape of spermathecae, but can be distinguished from the latter by abdominal disc with 33/34 ribs on each side (Fig. 3C).

#### Description.

Total length, including chelicerae, 22.50; chelicerae 3.40 long; carapace 9.70 long, 8.00 wide; abdomen 11.50 long, 14.20 wide. Carapace dark brown and smooth, with a few marginal hairs and a long bristle in the front of ocular area, three long bent bristles in longitudinal row running through ocular area (Fig. 3A). Carapace widest at coxae II. Ocular area black. Cervical groove and radial furrows distinct. Fovea deep and procurved, U-shaped, its greatest width occupying one fourth of carapace width at that point. Eyes set on low tubercle, ocular 1.00 long, 2.24 wide anteriorly, 2.20 wide posteriorly, occupying one fourth of carapace width at that point (Fig. 3A). Clypeus height 2.10. Both anterior and posterior eye rows straight and almost equal in length. Ratio of eyes, ALE: AME: PLE: PME (0.53: 0.34: 0.43: 0.33). ALE-AME 0.35, AME-AME 0.25, PLE-PME 0.05, PME-PME 1.00. MOA 1.00 long, 0.93 wide in front, 1.66 wide at back. Chelicerae red-brown, inner margin with eight teeth and six denticles between, outer margin with seven teeth and three denticles between. Rastellum raised on prominent angled projection and consisting of many short black teeth. Labium brown, 1.90 long, 1.70 wide, with three black cuspules anteriorly. Maxilla dark-brown, 18.5 long, 1.5 wide, with a few black cuspules at base. Sternum 5.50 long, 5.00 wide, with large, irregularly shaped sigilla in the centre (Fig. 3E).

Legs brown. Tibiae and tarsi of pedipalps, and distal three segments of legs I and II with numerous horn-like spines, metatarsus III with a few short dorsal ventrally spines, tarsus III with a few dark spines prolaterally and ventrally. Legs each with three tarsal claws, paired claws with a single large tooth, unpaired claw lacking tooth. Palp with a single claw bearing one tooth. Measurements: palp 14.50 (5.60 + 2.30 + 3.00 + 3.60), leg I 17.50 (6.00 + 3.00 + 3.70 + 2.80 + 2.00), leg II 14.40 (5.10 + 2.30 + 3.00 + 2.20 + 1.80), leg III 14.80 (5.20 + 3.00 + 2.30 + 2.20 + 2.10), leg IV 17.70 (5.50 + 3.20 + 3.50 + 3.10 + 2.40). Formula: 4132.

Abdomen funnel-shaped and dark brown. Caudal disc slightly convex (Fig. 3A, B), 13.20 in transversal diameter and 12.30 in longitudinal diameter, with two rids running dorso-ventrally (with small interrupt at the groove outer the upper pair muscle impressions) and 34/35 radiating ribs on each side (Fig. 3C). Abdominal disc with six well-marked muscle impressions, the middle pair muscle impression with an elevated central zone connected to the inner rim of muscle impression. Four spinnerets, with inner pair small and one-segmented, and outer pair much longer and three-segmented (Fig. 3D). Paired spermathecae sack-like, parallel-sided, the length of each one is more or less two times its width (Fig. 3F), length = 1.09 mm, width = 0.56 mm.

Male. Unknown.

#### Distribution.

China (Guizhou).

## Supplementary Material

XML Treatment for Cyclocosmia

XML Treatment for Cyclocosmia ricketti

XML Treatment for Cyclocosmia latusicosta

XML Treatment for Cyclocosmia liui
